# *N*,*N*′-Diacetyl-*N*′-[(4-nitro­phen­oxy)acetyl]acetohydrazide

**DOI:** 10.1107/S1600536809002232

**Published:** 2009-01-23

**Authors:** Xiao Hu, Zhifeng Wang, Weiren Xu, Guilong Zhao, Runling Wang

**Affiliations:** a School of Pharmacy, Tianjin Medical University, Tianjin 300070, People’s Republic of China; b Tianjin Key Laboratory of Molecular Design and Drug Discovery, Tianjin Institute of Pharmaceutical Research, Tianjin 300193, People’s Republic of China

## Abstract

The asymmetric unit of the title compound, C_14_H_15_N_3_O_7_, contains two independent mol­ecules which are linked into a pseudocentrosymmetric dimer by a π–π inter­action, as shown by the short distance of 3.722 (5) Å between the centroids of the benzene rings. An extensive network of weak inter­molecular C—H⋯O hydrogen bonds helps to stabilize the crystal packing.

## Related literature

For useful applications of hydrazide derivatives, see: Pang *et al.* (2005 ▶); Lutun *et al.* (1999 ▶); Liras *et al.* (2000 ▶); Dhadialla *et al.* (1998 ▶).
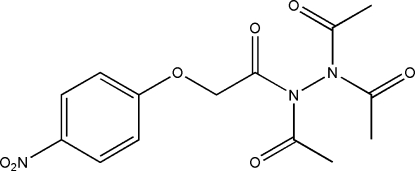


## Experimental

### 

#### Crystal data

C_14_H_15_N_3_O_7_
*M* *_r_* = 337.29
Monoclinic, 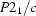

*a* = 14.949 (3) Å
*b* = 11.723 (2) Å
*c* = 23.034 (7) Å
β = 127.73 (2)°
*V* = 3192.6 (15) Å^3^
*Z* = 8
Mo *K*α radiation
μ = 0.11 mm^−1^
*T* = 113 (2) K
0.28 × 0.24 × 0.18 mm

#### Data collection

Rigaku Saturn diffractometer
Absorption correction: multi-scan (*CrystalClear*; Rigaku, 2005 ▶) *T*_min_ = 0.969, *T*_max_ = 0.980
21295 measured reflections
5619 independent reflections
2699 reflections with *I* > 2σ(*I*)
*R*_int_ = 0.082

#### Refinement

*R*[*F* ^2^ > 2σ(*F* ^2^)] = 0.057
*wR*(*F* ^2^) = 0.152
*S* = 0.90
5619 reflections
439 parameters
H-atom parameters constrained
Δρ_max_ = 0.19 e Å^−3^
Δρ_min_ = −0.24 e Å^−3^

### 

Data collection: *CrystalClear* (Rigaku, 2005 ▶); cell refinement: *CrystalClear*; data reduction: *CrystalClear*; program(s) used to solve structure: *SHELXTL* (Sheldrick, 2008 ▶); program(s) used to refine structure: *SHELXTL*; molecular graphics: *SHELXTL*; software used to prepare material for publication: *SHELXTL*.

## Supplementary Material

Crystal structure: contains datablocks I, global. DOI: 10.1107/S1600536809002232/cv2509sup1.cif

Structure factors: contains datablocks I. DOI: 10.1107/S1600536809002232/cv2509Isup2.hkl

Additional supplementary materials:  crystallographic information; 3D view; checkCIF report

## Figures and Tables

**Table 1:** Selected interatomic distances (Å) *Cg*1 and *Cg*2 are the centroids of the rings C1–C6 and C15–C20, respectively.

*Cg*1⋯*Cg*2	3.722 (5)

**Table 2:** Hydrogen-bond geometry (Å, °)

*D*—H⋯*A*	*D*—H	H⋯*A*	*D*⋯*A*	*D*—H⋯*A*
C16—H16*A*⋯O12^i^	0.93	2.52	3.328 (4)	146
C18—H18*A*⋯O13^ii^	0.93	2.46	3.214 (4)	138
C21—H21*B*⋯O7^iii^	0.97	2.48	3.422 (4)	165
C24—H24*A*⋯O3^iv^	0.96	2.41	3.194 (4)	139
C28—H28*C*⋯O8^iv^	0.96	2.51	3.465 (4)	172
C24—H24*C*⋯O14^v^	0.96	2.52	3.423 (4)	157
C26—H26*B*⋯O5^vi^	0.96	2.56	3.444 (4)	154
C10—H10*B*⋯O7^vii^	0.96	2.54	3.469 (4)	164
C12—H12*D*⋯O12^viii^	0.96	2.51	3.450 (4)	166

Symmetry codes: (i) 
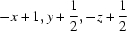
; (ii) 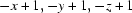; (iii) 
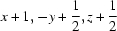
; (iv) 
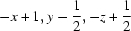
; (v) 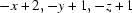; (vi) 
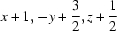
; (vii) 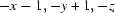; (viii) 
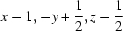
.

**Fig. 1.**
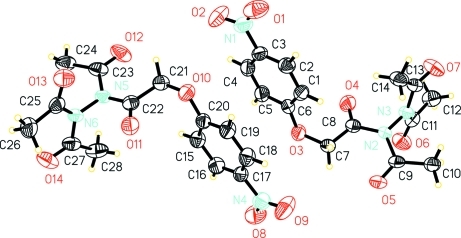
Two independent molecules of the title compound, with displacement ellipsoids drawn at the 40% probability level.

## supplementary crystallographic information

### Comment

Hydrazide derivates were extensively used in material, chemical and medical industy. *N*-(4-Alkoxybenzoyl)-*N*'-(4'-aminobenzoyl) hydrazine was used as a liquid crystalline material (Pang *et al.*, 2005) and bisacylhydrazines were a kind of insecticides (Dhadialla *et al.*, 1998). Hydrazide derivates were also important intermediates in the synthesis of 1,3,4-oxadiazole (Lutun *et al.*, 1999; Liras *et al.*, 2000), which was a significant heterocycle in chemical industy. In order to investigate its activity in anti-hyperglycaemia, we have synthesized the title compound.

The asymmetric unit of the title compound, C_14_H_15_N_3_O_7_, contains two independent molecules, which are linked into pseudo-centrosymmetric dimer by π–π interaction proved by short distance of 3.722 (5) Å between the centroids of benzene rings C1–C6 and C15–C20 (Table 1). An extensive network of weak intermolecular C—H···O hydrogen bonds (Table 2) help to stabilize the crystal packing.

### Experimental

1-Nitro-4-phenoxyacethydrazide (2.11 g, 0.01 mol) was dissolved in 30 ml of acetic anhydride. The solution was heated to reflux and stirred for 3 h and then cooled to room temperature. 300 ml of water were added and the precipitate formed was filtrated. The filter was wash with water and dried to give the title compound as a yellow powder (2.50 g, yield 76.4%, mp 400–402 K). The crystals suitable for the X-ray diffraction were obtained *via* slow evaporation of a solution of the title compound in dichloromethane/ethyl acetate/petroleum ether (1:1:1 v/v) at room temperature.

### Refinement

All H atoms were placed in calculated positions, with C—H = 0.93–0.97 Å, and included in the final cycles of refinement using a riding model, with *U*_iso_(H) = 1.2 (1.5 for methyl) times *U*_eq_(C).

### Crystal data

**Table:**

C_14_H_15_N_3_O_7_	*F*(000) = 1408
*M**_r_* = 337.29	*D*_x_ = 1.403 Mg m^−^^3^
Monoclinic, *P*2_1_/*c*	Mo *K*α radiation, λ = 0.71073 Å
Hall symbol: -P 2ybc	Cell parameters from 5002 reflections
*a* = 14.949 (3) Å	θ = 1.7–27.7°
*b* = 11.723 (2) Å	µ = 0.11 mm^−^^1^
*c* = 23.034 (7) Å	*T* = 113 K
β = 127.73 (2)°	Block, yellow
*V* = 3192.6 (15) Å^3^	0.28 × 0.24 × 0.18 mm
*Z* = 8	

### Data collection

**Table:**

Rigaku Saturn diffractometer	5619 independent reflections
Radiation source: rotating anode	2699 reflections with *I* > 2σ(*I*)
confocal	*R*_int_ = 0.082
ω scans	θ_max_ = 25.0°, θ_min_ = 3.1°
Absorption correction: multi-scan (*CrystalClear*; Rigaku, 2005)	*h* = −16→17
*T*_min_ = 0.969, *T*_max_ = 0.980	*k* = −13→13
21295 measured reflections	*l* = −25→27

### Refinement

**Table:**

Refinement on *F*^2^	Primary atom site location: structure-invariant direct methods
Least-squares matrix: full	Secondary atom site location: difference Fourier map
*R*[*F*^2^ > 2σ(*F*^2^)] = 0.057	Hydrogen site location: inferred from neighbouring sites
*wR*(*F*^2^) = 0.152	H-atom parameters constrained
*S* = 0.90	*w* = 1/[σ^2^(*F*_o_^2^) + (0.0651*P*)^2^] where *P* = (*F*_o_^2^ + 2*F*_c_^2^)/3
5619 reflections	(Δ/σ)_max_ = 0.001
439 parameters	Δρ_max_ = 0.19 e Å^−^^3^
0 restraints	Δρ_min_ = −0.24 e Å^−^^3^

### Special details

**Table:**

Geometry. All e.s.d.'s (except the e.s.d. in the dihedral angle between two l.s. planes) are estimated using the full covariance matrix. The cell e.s.d.'s are taken into account individually in the estimation of e.s.d.'s in distances, angles and torsion angles; correlations between e.s.d.'s in cell parameters are only used when they are defined by crystal symmetry. An approximate (isotropic) treatment of cell e.s.d.'s is used for estimating e.s.d.'s involving l.s. planes.
Refinement. Refinement of *F*^2^ against ALL reflections. The weighted *R*-factor *wR* and goodness of fit *S* are based on *F*^2^, conventional *R*-factors *R* are based on *F*, with *F* set to zero for negative *F*^2^. The threshold expression of *F*^2^ > σ(*F*^2^) is used only for calculating *R*-factors(gt) *etc*. and is not relevant to the choice of reflections for refinement. *R*-factors based on *F*^2^ are statistically about twice as large as those based on *F*, and *R*- factors based on ALL data will be even larger.

### Fractional atomic coordinates and isotropic or equivalent isotropic displacement parameters (Å^2^)

**Table:**

	*x*	*y*	*z*	*U*_iso_*/*U*_eq_	
O8	0.2333 (2)	0.8689 (2)	0.21267 (15)	0.0989 (9)	
O9	0.1368 (3)	0.8437 (2)	0.25247 (17)	0.1111 (11)	
O10	0.42854 (18)	0.40509 (18)	0.37921 (12)	0.0621 (6)	
O11	0.6321 (2)	0.49597 (19)	0.46900 (13)	0.0748 (7)	
O12	0.6482 (2)	0.2300 (2)	0.36527 (15)	0.0855 (8)	
O13	0.8291 (2)	0.3120 (2)	0.57451 (13)	0.0753 (7)	
O14	0.9409 (3)	0.5835 (2)	0.52505 (16)	0.0881 (8)	
N4	0.2110 (3)	0.8155 (2)	0.24781 (17)	0.0722 (8)	
N5	0.7227 (2)	0.38153 (19)	0.44137 (13)	0.0484 (6)	
N6	0.8218 (2)	0.43821 (19)	0.49755 (14)	0.0488 (6)	
C15	0.4096 (3)	0.5723 (3)	0.30951 (17)	0.0579 (9)	
H15A	0.4643	0.5466	0.3054	0.070*	
C16	0.3531 (3)	0.6740 (3)	0.27690 (17)	0.0606 (9)	
H16A	0.3689	0.7169	0.2502	0.073*	
C17	0.2740 (3)	0.7106 (3)	0.28447 (16)	0.0528 (8)	
C18	0.2493 (3)	0.6498 (3)	0.32446 (17)	0.0620 (9)	
H18A	0.1958	0.6767	0.3294	0.074*	
C19	0.3051 (3)	0.5486 (3)	0.35685 (18)	0.0591 (9)	
H19A	0.2902	0.5070	0.3844	0.071*	
C20	0.3834 (3)	0.5094 (3)	0.34818 (17)	0.0500 (8)	
C21	0.5136 (3)	0.3581 (3)	0.37718 (17)	0.0560 (9)	
H21A	0.4929	0.3675	0.3285	0.067*	
H21B	0.5209	0.2771	0.3879	0.067*	
C22	0.6249 (3)	0.4171 (3)	0.43298 (17)	0.0534 (8)	
C23	0.7331 (3)	0.2802 (3)	0.4132 (2)	0.0615 (10)	
C24	0.8493 (3)	0.2430 (3)	0.4436 (2)	0.0771 (11)	
H24A	0.8452	0.1809	0.4149	0.116*	
H24B	0.8886	0.2185	0.4935	0.116*	
H24C	0.8893	0.3055	0.4421	0.116*	
C25	0.8679 (3)	0.3978 (3)	0.56963 (19)	0.0574 (9)	
C26	0.9615 (3)	0.4641 (3)	0.6347 (2)	0.0828 (11)	
H26A	0.9821	0.4286	0.6789	0.124*	
H26B	0.9365	0.5407	0.6321	0.124*	
H26C	1.0259	0.4656	0.6349	0.124*	
C27	0.8542 (3)	0.5358 (3)	0.4788 (2)	0.0609 (9)	
C28	0.7769 (3)	0.5723 (3)	0.4003 (2)	0.0857 (12)	
H28A	0.8077	0.6384	0.3937	0.129*	
H28B	0.7040	0.5906	0.3872	0.129*	
H28C	0.7693	0.5116	0.3697	0.129*	
O1	0.1627 (3)	0.1506 (2)	0.30176 (15)	0.0978 (10)	
O2	0.2870 (3)	0.1469 (3)	0.28234 (18)	0.1256 (12)	
O3	0.04055 (18)	0.59236 (18)	0.11342 (13)	0.0692 (7)	
O4	−0.1592 (2)	0.50634 (19)	0.00605 (13)	0.0731 (7)	
O5	−0.2095 (2)	0.79848 (17)	0.07476 (13)	0.0723 (7)	
O6	−0.3611 (2)	0.6753 (2)	−0.12019 (13)	0.0814 (8)	
O7	−0.4705 (2)	0.4126 (2)	−0.06148 (13)	0.0832 (8)	
N1	0.2069 (3)	0.1901 (2)	0.27528 (17)	0.0771 (9)	
N2	−0.2660 (2)	0.62816 (18)	0.01591 (13)	0.0474 (6)	
N3	−0.3595 (2)	0.56424 (19)	−0.03922 (13)	0.0491 (6)	
C1	0.0295 (3)	0.4494 (3)	0.18604 (17)	0.0594 (9)	
H1A	−0.0283	0.4877	0.1823	0.071*	
C2	0.0735 (3)	0.3494 (3)	0.22626 (17)	0.0607 (9)	
H2A	0.0458	0.3202	0.2500	0.073*	
C3	0.1581 (3)	0.2942 (2)	0.23061 (17)	0.0561 (8)	
C4	0.2002 (3)	0.3346 (3)	0.19574 (19)	0.0661 (10)	
H4A	0.2574	0.2954	0.1993	0.079*	
C5	0.1565 (3)	0.4341 (3)	0.15526 (19)	0.0633 (9)	
H5A	0.1835	0.4620	0.1308	0.076*	
C6	0.0724 (3)	0.4919 (3)	0.15138 (18)	0.0547 (8)	
C7	−0.0556 (3)	0.6512 (3)	0.09652 (19)	0.0608 (9)	
H7A	−0.0561	0.7286	0.0815	0.073*	
H7B	−0.0529	0.6546	0.1397	0.073*	
C8	−0.1609 (3)	0.5901 (3)	0.03573 (18)	0.0528 (8)	
C9	−0.2871 (3)	0.7349 (2)	0.03504 (18)	0.0523 (8)	
C10	−0.4056 (3)	0.7574 (2)	0.00513 (19)	0.0625 (9)	
H10A	−0.4100	0.8297	0.0229	0.094*	
H10B	−0.4311	0.6982	0.0206	0.094*	
H10C	−0.4525	0.7590	−0.0475	0.094*	
C11	−0.4020 (3)	0.5946 (3)	−0.11249 (18)	0.0591 (9)	
C12	−0.4949 (3)	0.5243 (3)	−0.17484 (17)	0.0762 (11)	
H12A	−0.5187	0.5574	−0.2204	0.114*	
H12B	−0.5576	0.5222	−0.1733	0.114*	
H12D	−0.4681	0.4481	−0.1709	0.114*	
C13	−0.3882 (3)	0.4682 (2)	−0.01662 (19)	0.0534 (8)	
C14	−0.3147 (3)	0.4410 (3)	0.06307 (18)	0.0690 (10)	
H14D	−0.3470	0.3789	0.0717	0.103*	
H14A	−0.3088	0.5068	0.0901	0.103*	
H14B	−0.2408	0.4199	0.0789	0.103*	

### Atomic displacement parameters (Å^2^)

**Table:**

	*U*^11^	*U*^22^	*U*^33^	*U*^12^	*U*^13^	*U*^23^
O8	0.111 (2)	0.0792 (18)	0.105 (2)	0.0111 (17)	0.065 (2)	0.0332 (16)
O9	0.105 (2)	0.108 (2)	0.131 (3)	0.050 (2)	0.078 (2)	0.0477 (19)
O10	0.0612 (15)	0.0601 (14)	0.0769 (16)	0.0092 (12)	0.0483 (14)	0.0163 (12)
O11	0.0656 (17)	0.0729 (15)	0.0770 (17)	0.0062 (13)	0.0390 (15)	−0.0271 (14)
O12	0.093 (2)	0.0733 (16)	0.116 (2)	−0.0312 (16)	0.0768 (19)	−0.0495 (16)
O13	0.091 (2)	0.0692 (16)	0.0765 (18)	−0.0022 (15)	0.0569 (17)	0.0086 (13)
O14	0.097 (2)	0.0708 (17)	0.112 (2)	−0.0346 (16)	0.0712 (19)	−0.0268 (16)
N4	0.072 (2)	0.066 (2)	0.063 (2)	0.0024 (18)	0.0335 (19)	0.0085 (17)
N5	0.0546 (17)	0.0382 (13)	0.0590 (17)	−0.0047 (13)	0.0381 (15)	−0.0102 (12)
N6	0.0547 (17)	0.0396 (13)	0.0561 (17)	−0.0031 (13)	0.0359 (15)	−0.0048 (13)
C15	0.056 (2)	0.066 (2)	0.059 (2)	0.0020 (18)	0.039 (2)	0.0031 (18)
C16	0.063 (2)	0.064 (2)	0.055 (2)	−0.0002 (19)	0.036 (2)	0.0116 (17)
C17	0.049 (2)	0.0513 (19)	0.0444 (19)	0.0014 (16)	0.0213 (18)	0.0029 (15)
C18	0.059 (2)	0.074 (2)	0.063 (2)	0.005 (2)	0.042 (2)	0.0057 (19)
C19	0.058 (2)	0.067 (2)	0.064 (2)	0.0060 (19)	0.044 (2)	0.0117 (18)
C20	0.047 (2)	0.0522 (18)	0.0505 (19)	−0.0003 (16)	0.0300 (18)	0.0052 (16)
C21	0.062 (2)	0.0491 (18)	0.063 (2)	−0.0002 (18)	0.042 (2)	0.0004 (17)
C22	0.062 (2)	0.0481 (18)	0.055 (2)	0.0026 (18)	0.038 (2)	−0.0007 (17)
C23	0.086 (3)	0.0460 (18)	0.088 (3)	−0.009 (2)	0.071 (3)	−0.0146 (19)
C24	0.092 (3)	0.0503 (19)	0.126 (3)	0.000 (2)	0.086 (3)	−0.012 (2)
C25	0.055 (2)	0.054 (2)	0.065 (2)	0.0112 (19)	0.039 (2)	0.0003 (19)
C26	0.065 (3)	0.085 (3)	0.065 (2)	0.004 (2)	0.023 (2)	−0.005 (2)
C27	0.077 (3)	0.0436 (19)	0.083 (3)	−0.0058 (19)	0.060 (3)	−0.0097 (19)
C28	0.127 (4)	0.049 (2)	0.095 (3)	0.000 (2)	0.075 (3)	0.009 (2)
O1	0.134 (3)	0.0697 (18)	0.0722 (19)	0.0094 (17)	0.054 (2)	0.0135 (14)
O2	0.102 (3)	0.088 (2)	0.149 (3)	0.047 (2)	0.057 (2)	0.0269 (19)
O3	0.0543 (15)	0.0625 (14)	0.0942 (18)	0.0067 (12)	0.0472 (15)	0.0271 (13)
O4	0.0671 (17)	0.0672 (15)	0.0817 (17)	0.0038 (13)	0.0438 (15)	−0.0129 (14)
O5	0.0674 (17)	0.0409 (12)	0.0892 (19)	−0.0113 (12)	0.0381 (15)	−0.0120 (12)
O6	0.092 (2)	0.0794 (17)	0.0735 (18)	−0.0156 (15)	0.0506 (16)	0.0125 (14)
O7	0.088 (2)	0.0781 (17)	0.0704 (17)	−0.0390 (16)	0.0414 (16)	−0.0145 (14)
N1	0.079 (3)	0.0516 (19)	0.064 (2)	0.0040 (18)	0.025 (2)	−0.0032 (16)
N2	0.0470 (16)	0.0364 (13)	0.0561 (17)	−0.0079 (13)	0.0302 (15)	−0.0055 (12)
N3	0.0520 (17)	0.0420 (14)	0.0482 (16)	−0.0049 (13)	0.0280 (14)	−0.0015 (13)
C1	0.055 (2)	0.0529 (19)	0.074 (2)	0.0072 (17)	0.042 (2)	0.0103 (18)
C2	0.064 (2)	0.055 (2)	0.058 (2)	−0.0022 (19)	0.035 (2)	0.0048 (17)
C3	0.056 (2)	0.0420 (17)	0.051 (2)	0.0025 (17)	0.0231 (19)	−0.0017 (16)
C4	0.049 (2)	0.068 (2)	0.070 (2)	0.0018 (19)	0.030 (2)	−0.014 (2)
C5	0.053 (2)	0.066 (2)	0.069 (2)	0.0009 (19)	0.037 (2)	0.0060 (19)
C6	0.042 (2)	0.0493 (19)	0.062 (2)	−0.0029 (16)	0.0263 (18)	0.0018 (17)
C7	0.055 (2)	0.0425 (17)	0.078 (2)	−0.0018 (17)	0.037 (2)	0.0097 (17)
C8	0.058 (2)	0.0446 (18)	0.059 (2)	−0.0031 (18)	0.037 (2)	0.0046 (17)
C9	0.064 (2)	0.0340 (16)	0.060 (2)	−0.0035 (17)	0.038 (2)	0.0043 (16)
C10	0.069 (2)	0.0477 (18)	0.084 (3)	−0.0041 (18)	0.053 (2)	−0.0034 (18)
C11	0.060 (2)	0.059 (2)	0.058 (2)	0.0089 (19)	0.035 (2)	0.0037 (19)
C12	0.086 (3)	0.069 (2)	0.053 (2)	−0.002 (2)	0.031 (2)	−0.0051 (19)
C13	0.062 (2)	0.0412 (17)	0.061 (2)	−0.0066 (17)	0.039 (2)	−0.0028 (17)
C14	0.077 (3)	0.053 (2)	0.069 (2)	−0.0114 (18)	0.041 (2)	0.0077 (18)

### Geometric parameters (Å, °)

**Table:**

O8—N4	1.220 (3)	O1—N1	1.232 (4)
O9—N4	1.224 (4)	O2—N1	1.215 (4)
O10—C20	1.368 (3)	O3—C6	1.367 (3)
O10—C21	1.412 (3)	O3—C7	1.418 (3)
O11—C22	1.203 (3)	O4—C8	1.206 (3)
O12—C23	1.209 (4)	O5—C9	1.200 (3)
O13—C25	1.201 (4)	O6—C11	1.197 (4)
O14—C27	1.200 (4)	O7—C13	1.204 (3)
N4—C17	1.463 (4)	N1—C3	1.471 (4)
N5—N6	1.402 (3)	N2—N3	1.398 (3)
N5—C23	1.407 (4)	N2—C8	1.414 (4)
N5—C22	1.415 (4)	N2—C9	1.424 (4)
N6—C27	1.408 (4)	N3—C13	1.413 (3)
N6—C25	1.433 (4)	N3—C11	1.437 (4)
C15—C20	1.384 (4)	C1—C2	1.385 (4)
C15—C16	1.387 (4)	C1—C6	1.386 (4)
C15—H15A	0.9300	C1—H1A	0.9300
C16—C17	1.365 (4)	C2—C3	1.370 (4)
C16—H16A	0.9300	C2—H2A	0.9300
C17—C18	1.382 (4)	C3—C4	1.372 (4)
C18—C19	1.377 (4)	C4—C5	1.383 (4)
C18—H18A	0.9300	C4—H4A	0.9300
C19—C20	1.380 (4)	C5—C6	1.382 (4)
C19—H19A	0.9300	C5—H5A	0.9300
C21—C22	1.511 (4)	C7—C8	1.501 (4)
C21—H21A	0.9700	C7—H7A	0.9700
C21—H21B	0.9700	C7—H7B	0.9700
C23—C24	1.486 (4)	C9—C10	1.479 (4)
C24—H24A	0.9600	C10—H10A	0.9600
C24—H24B	0.9600	C10—H10B	0.9600
C24—H24C	0.9600	C10—H10C	0.9600
C25—C26	1.497 (5)	C11—C12	1.493 (4)
C26—H26A	0.9600	C12—H12A	0.9600
C26—H26B	0.9600	C12—H12B	0.9600
C26—H26C	0.9600	C12—H12D	0.9600
C27—C28	1.494 (5)	C13—C14	1.487 (4)
C28—H28A	0.9600	C14—H14D	0.9600
C28—H28B	0.9600	C14—H14A	0.9600
C28—H28C	0.9600	C14—H14B	0.9600
			
Cg1···Cg2	3.722 (5)		
			
C20—O10—C21	119.9 (2)	C6—O3—C7	119.5 (2)
O8—N4—O9	123.3 (3)	O2—N1—O1	124.1 (4)
O8—N4—C17	118.3 (3)	O2—N1—C3	117.8 (4)
O9—N4—C17	118.4 (3)	O1—N1—C3	118.1 (4)
N6—N5—C23	118.2 (3)	N3—N2—C8	114.8 (2)
N6—N5—C22	114.0 (2)	N3—N2—C9	117.7 (2)
C23—N5—C22	125.6 (3)	C8—N2—C9	126.1 (3)
N5—N6—C27	118.0 (3)	N2—N3—C13	116.9 (2)
N5—N6—C25	113.6 (2)	N2—N3—C11	114.3 (2)
C27—N6—C25	127.6 (3)	C13—N3—C11	127.9 (3)
C20—C15—C16	119.2 (3)	C2—C1—C6	119.5 (3)
C20—C15—H15A	120.4	C2—C1—H1A	120.3
C16—C15—H15A	120.4	C6—C1—H1A	120.3
C17—C16—C15	119.2 (3)	C3—C2—C1	119.2 (3)
C17—C16—H16A	120.4	C3—C2—H2A	120.4
C15—C16—H16A	120.4	C1—C2—H2A	120.4
C16—C17—C18	121.9 (3)	C2—C3—C4	121.9 (3)
C16—C17—N4	119.5 (3)	C2—C3—N1	119.0 (3)
C18—C17—N4	118.6 (3)	C4—C3—N1	119.2 (3)
C19—C18—C17	119.0 (3)	C3—C4—C5	119.3 (3)
C19—C18—H18A	120.5	C3—C4—H4A	120.3
C17—C18—H18A	120.5	C5—C4—H4A	120.3
C18—C19—C20	119.6 (3)	C6—C5—C4	119.5 (3)
C18—C19—H19A	120.2	C6—C5—H5A	120.2
C20—C19—H19A	120.2	C4—C5—H5A	120.2
O10—C20—C19	114.4 (3)	O3—C6—C5	114.2 (3)
O10—C20—C15	124.6 (3)	O3—C6—C1	125.1 (3)
C19—C20—C15	121.0 (3)	C5—C6—C1	120.6 (3)
O10—C21—C22	109.8 (2)	O3—C7—C8	109.4 (3)
O10—C21—H21A	109.7	O3—C7—H7A	109.8
C22—C21—H21A	109.7	C8—C7—H7A	109.8
O10—C21—H21B	109.7	O3—C7—H7B	109.8
C22—C21—H21B	109.7	C8—C7—H7B	109.8
H21A—C21—H21B	108.2	H7A—C7—H7B	108.2
O11—C22—N5	118.9 (3)	O4—C8—N2	118.6 (3)
O11—C22—C21	121.8 (3)	O4—C8—C7	123.0 (3)
N5—C22—C21	119.3 (3)	N2—C8—C7	118.3 (3)
O12—C23—N5	118.8 (3)	O5—C9—N2	119.2 (3)
O12—C23—C24	124.0 (3)	O5—C9—C10	124.6 (3)
N5—C23—C24	117.2 (3)	N2—C9—C10	116.2 (3)
C23—C24—H24A	109.5	C9—C10—H10A	109.5
C23—C24—H24B	109.5	C9—C10—H10B	109.5
H24A—C24—H24B	109.5	H10A—C10—H10B	109.5
C23—C24—H24C	109.5	C9—C10—H10C	109.5
H24A—C24—H24C	109.5	H10A—C10—H10C	109.5
H24B—C24—H24C	109.5	H10B—C10—H10C	109.5
O13—C25—N6	117.9 (3)	O6—C11—N3	118.1 (3)
O13—C25—C26	123.4 (3)	O6—C11—C12	123.5 (3)
N6—C25—C26	118.8 (3)	N3—C11—C12	118.4 (3)
C25—C26—H26A	109.5	C11—C12—H12A	109.5
C25—C26—H26B	109.5	C11—C12—H12B	109.5
H26A—C26—H26B	109.5	H12A—C12—H12B	109.5
C25—C26—H26C	109.5	C11—C12—H12D	109.5
H26A—C26—H26C	109.5	H12A—C12—H12D	109.5
H26B—C26—H26C	109.5	H12B—C12—H12D	109.5
O14—C27—N6	119.7 (3)	O7—C13—N3	119.8 (3)
O14—C27—C28	123.7 (3)	O7—C13—C14	122.6 (3)
N6—C27—C28	116.6 (3)	N3—C13—C14	117.6 (3)
C27—C28—H28A	109.5	C13—C14—H14D	109.5
C27—C28—H28B	109.5	C13—C14—H14A	109.5
H28A—C28—H28B	109.5	H14D—C14—H14A	109.5
C27—C28—H28C	109.5	C13—C14—H14B	109.5
H28A—C28—H28C	109.5	H14D—C14—H14B	109.5
H28B—C28—H28C	109.5	H14A—C14—H14B	109.5
			
C23—N5—N6—C27	102.4 (3)	C8—N2—N3—C13	87.8 (3)
C22—N5—N6—C27	−93.4 (3)	C9—N2—N3—C13	−104.8 (3)
C23—N5—N6—C25	−86.8 (3)	C8—N2—N3—C11	−82.3 (3)
C22—N5—N6—C25	77.4 (3)	C9—N2—N3—C11	85.1 (3)
C20—C15—C16—C17	0.8 (5)	C6—C1—C2—C3	−0.4 (5)
C15—C16—C17—C18	0.8 (5)	C1—C2—C3—C4	−0.5 (5)
C15—C16—C17—N4	−177.6 (3)	C1—C2—C3—N1	178.1 (3)
O8—N4—C17—C16	−1.7 (5)	O2—N1—C3—C2	−175.2 (3)
O9—N4—C17—C16	176.6 (3)	O1—N1—C3—C2	6.5 (5)
O8—N4—C17—C18	179.9 (3)	O2—N1—C3—C4	3.5 (5)
O9—N4—C17—C18	−1.8 (5)	O1—N1—C3—C4	−174.8 (3)
C16—C17—C18—C19	−0.8 (5)	C2—C3—C4—C5	0.3 (5)
N4—C17—C18—C19	177.5 (3)	N1—C3—C4—C5	−178.3 (3)
C17—C18—C19—C20	−0.7 (5)	C3—C4—C5—C6	0.8 (5)
C21—O10—C20—C19	−177.0 (3)	C7—O3—C6—C5	171.0 (3)
C21—O10—C20—C15	4.4 (4)	C7—O3—C6—C1	−11.1 (5)
C18—C19—C20—O10	−176.4 (3)	C4—C5—C6—O3	176.4 (3)
C18—C19—C20—C15	2.3 (5)	C4—C5—C6—C1	−1.7 (5)
C16—C15—C20—O10	176.2 (3)	C2—C1—C6—O3	−176.4 (3)
C16—C15—C20—C19	−2.3 (5)	C2—C1—C6—C5	1.5 (5)
C20—O10—C21—C22	75.9 (3)	C6—O3—C7—C8	−74.3 (3)
N6—N5—C22—O11	5.7 (4)	N3—N2—C8—O4	−1.9 (4)
C23—N5—C22—O11	168.5 (3)	C9—N2—C8—O4	−168.2 (3)
N6—N5—C22—C21	−176.6 (2)	N3—N2—C8—C7	−177.7 (2)
C23—N5—C22—C21	−13.8 (4)	C9—N2—C8—C7	16.1 (4)
O10—C21—C22—O11	−3.7 (4)	O3—C7—C8—O4	−2.3 (4)
O10—C21—C22—N5	178.7 (2)	O3—C7—C8—N2	173.3 (2)
N6—N5—C23—O12	179.3 (3)	N3—N2—C9—O5	−170.1 (2)
C22—N5—C23—O12	17.1 (5)	C8—N2—C9—O5	−4.2 (4)
N6—N5—C23—C24	−2.2 (4)	N3—N2—C9—C10	11.6 (4)
C22—N5—C23—C24	−164.4 (3)	C8—N2—C9—C10	177.5 (3)
N5—N6—C25—O13	10.5 (4)	N2—N3—C11—O6	−5.7 (4)
C27—N6—C25—O13	−179.8 (3)	C13—N3—C11—O6	−174.6 (3)
N5—N6—C25—C26	−169.5 (3)	N2—N3—C11—C12	174.5 (3)
C27—N6—C25—C26	0.3 (4)	C13—N3—C11—C12	5.7 (4)
N5—N6—C27—O14	−177.0 (3)	N2—N3—C13—O7	177.2 (3)
C25—N6—C27—O14	13.7 (5)	C11—N3—C13—O7	−14.3 (5)
N5—N6—C27—C28	2.0 (4)	N2—N3—C13—C14	−2.1 (4)
C25—N6—C27—C28	−167.4 (3)	C11—N3—C13—C14	166.4 (3)

### Hydrogen-bond geometry (Å, °)

**Table:**

*D*—H···*A*	*D*—H	H···*A*	*D*···*A*	*D*—H···*A*
C16—H16A···O12^i^	0.93	2.52	3.328 (4)	146
C18—H18A···O13^ii^	0.93	2.46	3.214 (4)	138
C21—H21B···O7^iii^	0.97	2.48	3.422 (4)	165
C24—H24A···O3^iv^	0.96	2.41	3.194 (4)	139
C28—H28C···O8^iv^	0.96	2.51	3.465 (4)	172
C24—H24C···O14^v^	0.96	2.52	3.423 (4)	157
C26—H26B···O5^vi^	0.96	2.56	3.444 (4)	154
C10—H10B···O7^vii^	0.96	2.54	3.469 (4)	164
C12—H12D···O12^viii^	0.96	2.51	3.450 (4)	166

Symmetry codes: (i) −*x*+1, *y*+1/2, −*z*+1/2; (ii) −*x*+1, −*y*+1, −*z*+1; (iii) *x*+1, −*y*+1/2, *z*+1/2; (iv) −*x*+1, *y*−1/2, −*z*+1/2; (v) −*x*+2, −*y*+1, −*z*+1; (vi) *x*+1, −*y*+3/2, *z*+1/2; (vii) −*x*−1, −*y*+1, −*z*; (viii) *x*−1, −*y*+1/2, *z*−1/2.
